# Reduction of the occlusion effect induced by earplugs using quasi perfect broadband absorption

**DOI:** 10.1038/s41598-022-19641-3

**Published:** 2022-09-12

**Authors:** Kévin Carillo, Franck Sgard, Olivier Dazel, Olivier Doutres

**Affiliations:** 1 École de Technologie Superieure (ETS), Montréal, QC Canada; 2 Institut de Recherche Robert-Sauvé en Santé et en Sécurité du Travail (IRSST), Montréal, QC Canada; 3 Laboratoire d’Acoustique de l’Université du Mans (LAUM), UMR 6613, Institut d’Acoustique – Graduate School (IA-GS), CNRS, Le Mans Université, Le Mans, France

**Keywords:** Acoustics, Risk factors

## Abstract

Passive earplugs are used to prevent workers from noise-induced hearing loss. However, earplugs often induce an acoustic discomfort known as the occlusion effect. This phenomenon corresponds to an increased auditory perception of the bone-conducted part of physiological noises at low-frequency and is associated with the augmentation of the acoustic pressure in the occluded earcanal. In this work, we report a new concept of passive earplugs for mitigating the occlusion effect between 100 Hz and 1 kHz. The strategy consists in reducing the input impedance of the earplug seen from the earcanal by using quasi-perfect broadband absorbers derived from the field of meta-materials. The proposed “meta-earplug” is made of 4 critically coupled Helmholtz resonators arranged in parallel. Their geometry is optimized using an evolutionary algorithm associated with a theoretical model of the meta-earplug input impedance. The latter is validated against a finite-element approach and impedance sensor measurements. The meta-earplug is manufactured by 3D printing. Artificial test fixtures are used to assess the occlusion effect and the insertion loss. Results show that the meta-earplug induces an occlusion effect approximately 10 dB lower than foam and silicone earplugs while it provides an insertion loss similar to the silicone earplug up to 5 kHz.

## Introduction

Among the many causes of hearing loss, occupational noise represents an important risk factor^1^. Hearing protection devices such as earmuffs and earplugs are commonly used in noisy environment to protect workers from noise-induced hearing loss (NIHL). However, the lack of comfort associated with wearing hearing protectors strongly affects their use and thus their efficiency for preventing NIHL^2,3^. Earplug comfort is a multidimensional construct that encompasses physical, functional, acoustical and psychological aspects^2^. Regarding the acoustical dimension, the use of earplugs is usually associated with the occlusion effect^3,4^. This phenomenon is described as an uncomfortable increased auditory perception of the bone-conducted part of one’s own physiological noise (e.g., one’s own voice, chewing, breathing, etc.) when the earcanal entrance is covered or blocked, and is most significant at low frequencies, typically below 1 kHz^3^.

The sensation of the occlusion effect is objectively associated with an augmentation of the acoustic pressure in the occluded earcanal compared to the case where the earcanal is open, i.e., not obstructed by the in-ear device^5^. Under a bone-conducted stimulation (e.g., vocal cords, bone-transducer, etc.), the earcanal wall vibrates and generates an acoustic pressure in the earcanal cavity which depends on the open or occluded state of the entrance. At low frequencies, the acoustic impedance (seen by the earcanal wall) of the earcanal cavity occluded by earplugs is governed by the compressibility effect of the occluded volume and is significantly higher than the acoustic impedance of the open earcanal which is rather governed by its inertia effect. This change in character of the acoustic impedance of the earcanal seen by its wall corresponds to the fundamental mechanism of the objective occlusion effect^6^. Note that the medial surface of earplugs also vibrates due to the bone-conducted stimulation and contributes together with the earcanal wall to the acoustic pressure generated in the occluded earcanal^7^.

The deep insertion of the occlusion device can be used to reduce the occlusion effect induced by earplugs as well as hearing aids^8,9^. This solution is based on the reduction of the vibrating earcanal wall area generating acoustic pressure in the occluded earcanal. However, the deep insertion can be responsible for mechanical discomfort due to the sensitivity of the earcanal bony part. For hearing aids, the use of vents^10–12^ or open fittings^13,14^ can drastically reduce the occlusion effect. These solutions are based on the reduction of the acoustic impedance of the occluded earcanal compared to an acoustically rigid occlusion^15^ but come at the cost of decreasing the hearing aid performance due to acoustic feedback, limited amplification gain and near zero suppression of ambient noise^12,16^. Furthermore, these solutions are not suitable for earplugs to ensure sufficient sound attenuation required for hearing protection purpose. More recently, active systems reducing the occlusion effect of earplugs^17^, hearing aids^18,19^ and earbuds^16^ have been developed based on the principle of destructive interference. These systems have a great potential to obtain a natural perception of one’s own voice^16^ but can be subjected to the generation of distorted sounds when the sound pressure level in the occluded earcanal exceeds the maximum output level of the active noise cancelation system^19^. In addition, while these systems are not yet available on the market to the authors’ knowledge, they are likely be more expensive than passive solutions. Hence, new passive solutions are sought to reduce the occlusion effect for improving the acoustic comfort of earplugs’ users.

To mitigate the occlusion effect induced by a passive earplug, we propose here another solution than existing venting or open-fitting systems. This solution consists in modifying the earplug medial surface acoustic impedance so that it matches the characteristic impedance of air in order to reduce the acoustic impedance seen by the earcanal walls. This is achieved by using quasi-perfect broadband absorbers developed in the field of acoustic meta-materials^20,21^ and which are adapted here to the design of the first “meta-earplug”. The quasi-perfect broadband absorption can be achieved by using critically coupled resonant systems arranged in series or parallel^20–22^. The critical coupling corresponds to the perfect balance between the energy leakage from the resonator to the environment and the energy dissipation in the resonator, which leads to the perfect absorption. Compared to the use of vents, the proposed solution allows for reducing the objective occlusion effect while ensuring a sound attenuation adapted to hearing protection purposes.

**Figure 1:**
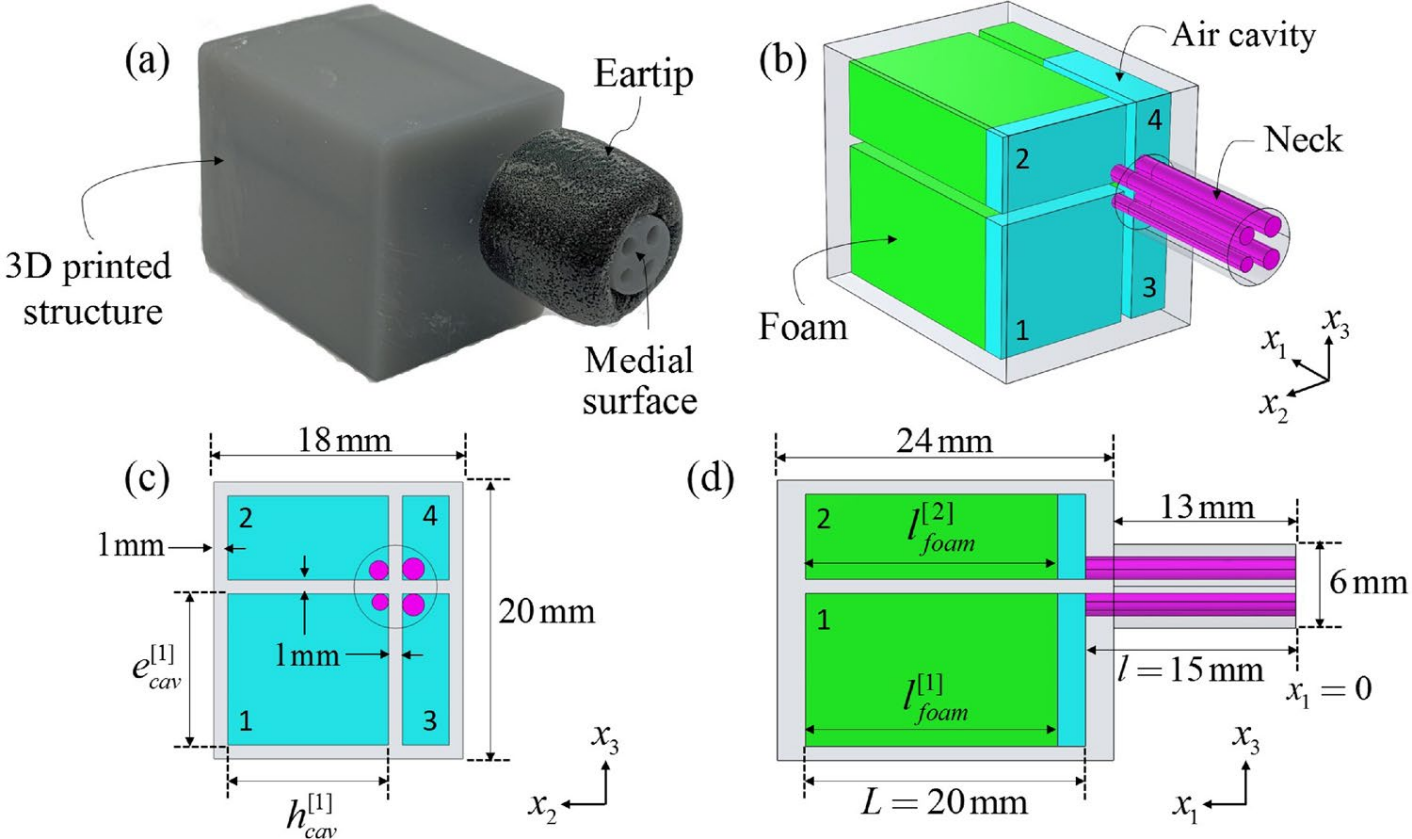
(**a**) 3D printed meta-earplug and schematics of the meta-earplug in (**b**) 3D view and (**c,d**) 2D planes. Numbers 1 to 4 identify each HR.

The meta-earplug presented here and displayed in Fig. 1 is made of 4 critically coupled Helmholtz resonators (HRs) arranged in parallel. Each HR includes a neck and a cavity which is partially filled by an acoustic foam. Necks of resonators are gathered in a thin cylindrical structure that is inserted in the earcanal cartilaginous part (around 10 mm insertion depth). This structure is surrounded by a Comply® foam eartip which can adapt to various earcanal size and seal the entrance to ensure sound attenuation and maintain the meta-earplug in position. The cavities of HRs are included in a parallelepipedic volume constrained to approximately 7 cm^3^. Hence, cavities of the meta-earplug could partially fit in the concha (around 4 cm^3^^23^) and slightly protrude outside the pinna like commercial earbuds including active noise cancellation device. In this paper, the geometry of the meta-earplug was kept simple (i.e., not adapted to real pinna) to focus on the understanding of its vibro-acoustic behaviour and its abilities to reduce the occlusion effect while providing a suitable sound attenuation.

The optimization process of the geometry of the meta-earplug was based on an evolutionary algorithm^24^ associated with a theoretical model of the reflection coefficient $R$ of its medial surface. The cost function used in the optimization process was $\varepsilon ={\int }_{{f}_{init}}^{{f}_{end}}|R(f){|}^{2}\mathrm{d}f$, where $f$ is the frequency, to minimize the reflection coefficient in a broadband frequency range starting from ${f}_{init}=200$ Hz to ${f}_{end}=900$ Hz. This covers most of the frequencies where the occlusion effect is significant. Two geometrical parameters (${e}_{cav}^{[1]}$ and ${h}_{cav}^{[1]}$) define the topology of the resonators and, for each resonator, the thickness of the foam layer (${l}_{foam}$) and the radius of the neck (${r}_{neck}$) are optimized. Geometrical parameters resulting from the optimization process are summarized in Table 1.

**Table 1:** Geometrical parameters (in mm) obtained by the optimization process for the meta-earplug.

${r}_{neck}^{[1]}$	${r}_{neck}^{[2]}$	${r}_{neck}^{[3]}$	${r}_{neck}^{[4]}$	${e}_{cav}^{[1]}$	${h}_{cav}^{[1]}$	${l}_{foam}^{[1]}$	${l}_{foam}^{[2]}$	${l}_{foam}^{[3]}$	${l}_{foam}^{[4]}$
$0.6$	$0.7$	$0.8$	$0.8$	$10.9$	$11.6$	$18$	$18$	$18$	$8.4$

Superscripts 1 to 4 refer to each HR.

In the following, the acoustic properties of the meta-earplug medial surface as well as the occlusion effect and the sound attenuation induced by the meta-earplug are investigated and discussed.

## Results

### Acoustic properties of the meta-earplug medial surface

**Figure 2:**
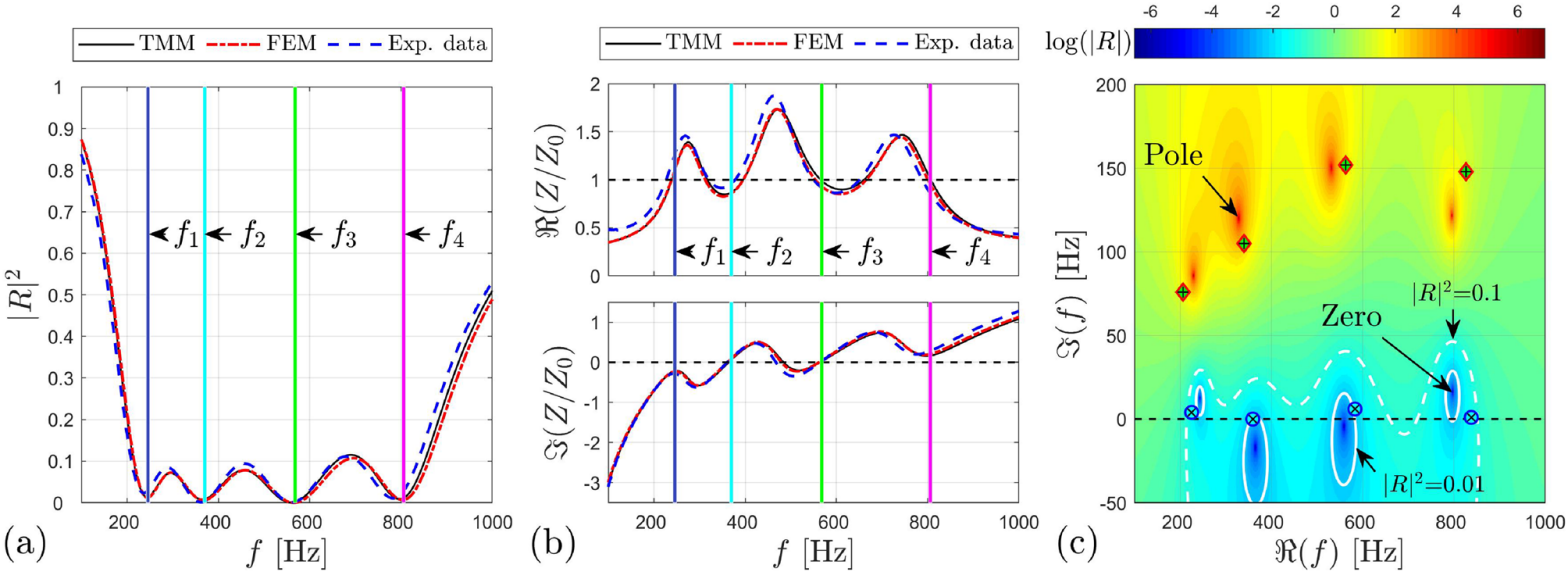
(**a**) Reflection coefficient and (**b**) normalized acoustic impedance of the meta-earplug of medial surface ${S}_{EC}$ calculated analytically (TMM), numerically (FEM) and measured experimentally using an impedance sensor^25^. Vertical coloured lines indicate frequencies of absorption peaks of the meta-earplug, i.e., ${f}_{1}=245$ Hz, ${f}_{2}=367$ Hz, ${f}_{3}=565$ Hz, and ${f}_{4}=803$ Hz. (**c**) Reflection coefficient of the meta-earplug in the complex frequency plane analytically computed using the TMM. White continuous (respectively dashed) lines show the isoline $|R{|}^{2}=0.01$ (respectively $|R{|}^{2}=0.1$). Diamond and circle symbols represent poles and zeros of HRs isolated from each other (i.e., taken individually). Symbols $\mathfrak{R}$ and $\mathfrak{I}$ represent real and imaginary part respectively.

We start by investigating the acoustic behaviour of the meta-earplug medial surface of area ${S}_{EC}=\pi {r}_{EC}^{2}$ where ${r}_{EC}=3.75$ mm corresponds to an average earcanal radius used in artificial test fixtures (see “Occlusion effect” and “Insertion loss” sections). Figure 2a,b displays the corresponding reflection coefficient and normalized acoustic impedance (real and imaginary parts) calculated (i) analytically using the transfer matrix method (TMM), (ii) numerically using the finite element method (FEM), and (iii) experimentally measured using an impedance sensor^25^. Note that for the measurements, the meta-earplug included a built-in 3D printed support adapted to the impedance sensor (see Fig. 6b) rather than the foam eartip seen in Fig. 1a. All the methodological details regarding the analytical and numerical models and the measurement are provided in “Methods” section. According to Fig. 2a,b, good agreement is observed between analytical, numerical, and experimental results. Slight discrepancies exist between models and experimental data due to inherent inaccuracies of both the 3D printing process and the melamine foam cutting.

Figure 2a shows that the reflection coefficient of the meta-earplug medial surface is lower than 0.1 between 200 and 900 Hz. This frequency range corresponds to that defined in the optimization process to cover the frequency region where the occlusion effect is most significant. Assuming negligible sound transmission through the 3D printed structure of the meta-earplug, the relation $\alpha =1-|R{|}^{2}$, where $\alpha$ is the absorption coefficient, holds. Hence, the meta-earplug medial surface exhibits a quasi-perfect broadband absorption between 200 and 900 Hz. In Fig. 2a, Vertical coloured lines indicate frequencies of absorption peaks of the meta-earplug, i.e., ${f}_{1}=245$ Hz, ${f}_{2}=367$ Hz, ${f}_{3}=565$ Hz, and ${f}_{4}=803$ Hz (obtained by graphic reading), respectively governed by HR#1, HR#2, HR#3 and HR#4.

As presented in the introduction, the aim for obtaining a quasi-perfect broadband absorption of the meta-earplug medial surface is to match the characteristic impedance of air in order to reduce the occlusion effect. This will be investigated in “Occlusion effect” section. Here, Fig. 2b shows that, at the absorption peaks of the meta-earplug (i.e., ${f}_{1}$–${f}_{4}$), the real part of the normalized impedance is close to 1 while its imaginary part is almost 0. Hence, the acoustic impedance of the meta-earplug medial surface is mainly resistive and fulfils the conditions of impedance matching. On the contrary, below 200 Hz and above 900 Hz, the real part of the normalized impedance vanishes while its imaginary part increases. The acoustic impedance of the meta-earplug medial surface becomes mainly reactive and departs from the impedance matching conditions.

The perfect absorption behaviour that supports the impedance matching of the meta-earplug medial surface with the air environment originates from the balance between the energy leakage from the HRs to the main waveguide (i.e., the earcanal) and the energy dissipated in the HRs^20^. When evaluating the reflection coefficient in the complex frequency plane, the balance between energy leakage and energy dissipation is achieved when the zeros of the function are located on the real frequency axis^20,21^. Figure 2c displays the reflection coefficient of the meta-earplug calculated in the complex frequency plane using the TMM model. The zeros are not exactly located on the real frequency axis but close enough to provide quasi-perfect absorption. The sign of the imaginary part of the zeros indicates if the perfect absorption is not achieved due to a lack of losses (i.e., negative imaginary part) or an excess (i.e., positive imaginary part)^20^. Regarding the poles, their imaginary part is related to the leakage rate of energy from the HRs to the waveguide and the distance between a given pole and its associated zero gives information on the bandwidth of the absorption peak^20^: the greater the distance, the wider the absorption peak. Since HR#3 presents a wider absorption peak than other HRs (see the distance between HR#3’s pole and zero in Fig. 2c), it ensures a larger frequency band of quasi-perfect absorption (see the white continuous line crossing the real frequency axis at ${f}_{3}=565$ Hz in Fig. 2c) and can be located further from its neighbours HR#2 and HR#4 than HR#1 and HR#2 are, while preserving a quasi-perfect broadband absorption of the meta-earplug medial surface.

The representation of the reflection coefficient in the complex frequency plane is also interesting to study the interaction of the HRs of the meta-earplug through their interference in the waveguide^20^. For this purpose, Fig. 2c also displays the pole (red diamond symbols) and associated zero (blue circle symbols) of each HR taken individually (i.e., HRs isolated from each other). We can see that position of poles and zeros in the complex frequency plane are slightly changed from coupled HRs to isolated HRs. Hence, frequencies of absorption peaks of the meta-earplug medial surface do not exactly correspond to resonance frequencies of HRs due to their coupling through the waveguide (i.e., which will be the earcanal in practice). According to Fig. 2c, zeros of isolated HRs are almost all located on the real frequency axis. This corresponds to a quasi-critical coupling of each individual HR which supports the quasi-perfect broadband absorption of the meta-earplug. Compared to the coupled system, the distance between pole and zero of uncoupled HRs is only governed by the change in section between their neck and the main waveguide. The larger this change, the lesser the distance between their respective poles and zeros. This can be seen on Fig. 2c where HR#3 and HR#4 have the largest neck radius (both equal to 0.8 mm). In consequence, they also have the largest absorption bandwidth (see Supplementary Materials).

**Figure 3:**
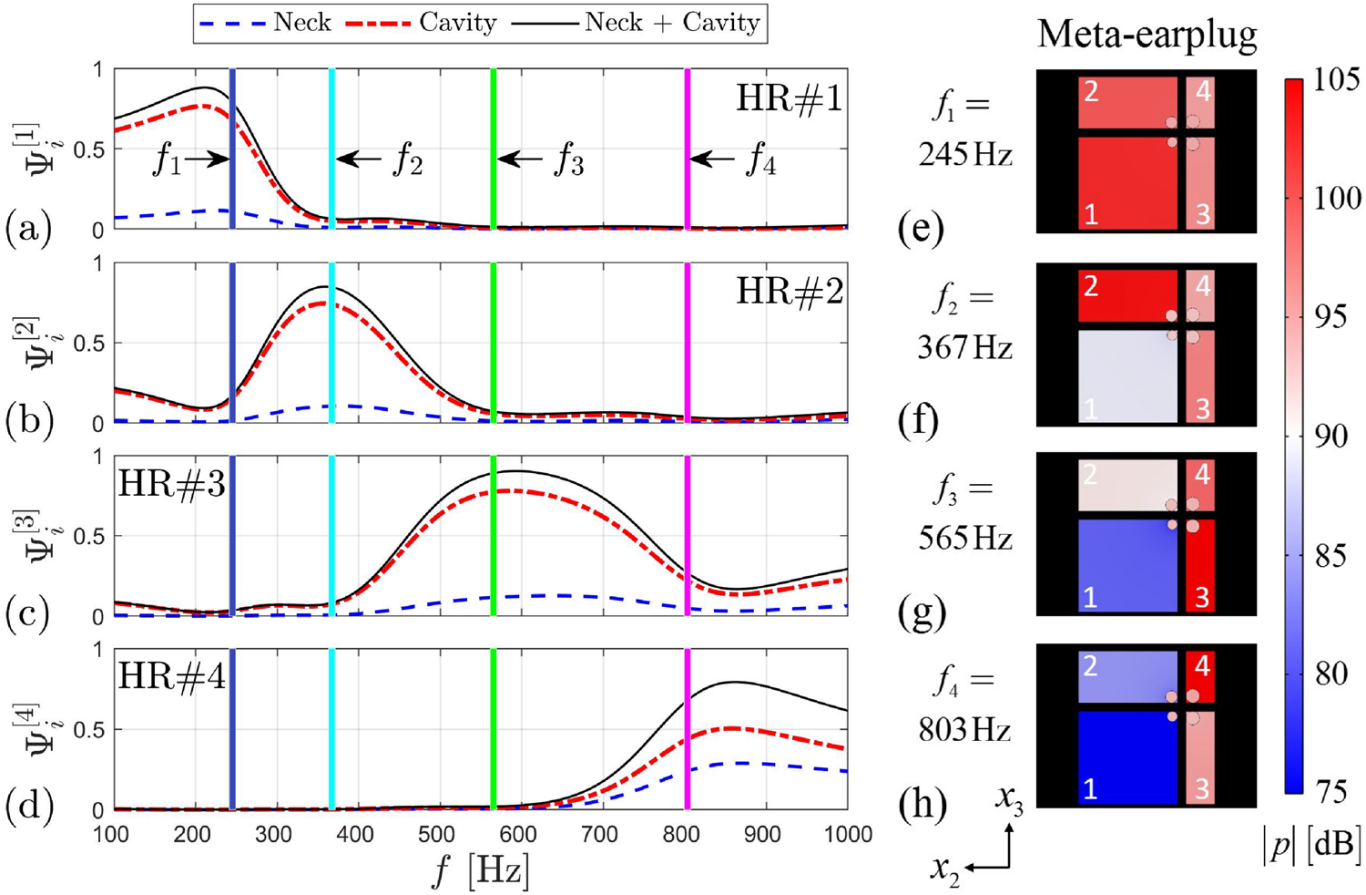
(**a–d**) Acoustic power ${\overline{W} }_{i}^{[n]}$ (time-averaged over one period) dissipated in neck and cavity for each HR in proportion of the total acoustic power ${\overline{W} }_{tot}$ dissipated in the meta-earplug (${\Psi }_{i}^{[n]}={\overline{W} }_{i}^{[n]}/{\overline{W} }_{tot}$, where $i\in \left\{neck ; cavity ; neck+cavity\right\}$ and $n\in \left\{1 ; 2 ; 3 ; 4\right\}$ the index of each HR). In each cavity domain, the foam layer governs the acoustic dissipation. Vertical coloured lines indicate frequencies of absorption peaks of the meta-earplug, i.e., ${f}_{1}=245$ Hz, ${f}_{2}=367$ Hz, ${f}_{3}=565$ Hz, and ${f}_{4}=803$ Hz. (**e–h**) Acoustic pressure fields inside each resonator at frequencies of absorption peaks of the meta-earplug (colour-map in $20{\mathrm{log}}_{10}\left(\left|p/{p}_{ref}\right|\right)$, where ${p}_{ref}=2\times 1{0}^{-5}$ Pa). Results were computed using the numerical model.

To further investigate the contribution of each HR to the quasi-perfect broadband absorption of the meta-earplug, Fig. 3a–d displays, for each HR, the proportion of acoustic power dissipated in the HR (neck, cavity including foam layer and both neck and cavity) relatively to the total acoustic power dissipated in the meta-earplug. Vertical coloured lines indicate frequencies of absorption peaks of the meta-earplug (i.e., ${f}_{1}$–${f}_{4}$). Results were computed using the numerical model by integrating the total dissipated (by visco-thermal effects) acoustic power density ${Q}_{pw}= -2|\mathbf{I}|\mathfrak{I}(k)$ over the volumes of each HR components, where $|\mathbf{I}|$ is the acoustic intensity vector magnitude and $k$ the acoustic wavenumber in neck and cavity (including foam) volumes of each HR. First, we can see that the dissipation of acoustic energy mainly occurs in the cavities of HRs due to the melamine foam layers inserted to achieve total absorption of sound. In Supplementary Materials, we also show that the melamine foam shifts towards lower frequencies the acoustic resonance of each HR by increasing the acoustic compliance of the cavities. Hence, for a given cavity volume, the foam allows for reaching the perfect absorption behaviour at lower frequencies. The dissipation in the necks of HRs is of secondary importance but also contributes to the quasi-perfect broadband absorption of the meta-earplug medial surface. Second, Fig. 3a–d shows that absorption peaks of the meta-earplug do not exactly happen at the uncoupled resonance frequencies of HRs, when the dissipation provided by each of them is maximum. Indeed, absorption peaks of the meta-earplug are slightly influenced by the coupling between HRs, as observed in the complex frequency plane (see Fig. 2c). To illustrate the coupling between HRs, Fig. 3e–h displays the acoustic pressure field in the meta-earplug computed at the absorption peaks frequencies (i.e., ${f}_{1}$–${f}_{4}$). At each of these frequencies of absorption peaks, we can see that the acoustic pressure is maximum in the HR of closest resonance frequency but other HRs are also slightly activated. These couplings between HRs also contribute to produce the quasi-perfect broadband absorption achieved by the meta-earplug medial surface.

### Occlusion effect

**Figure 4:**
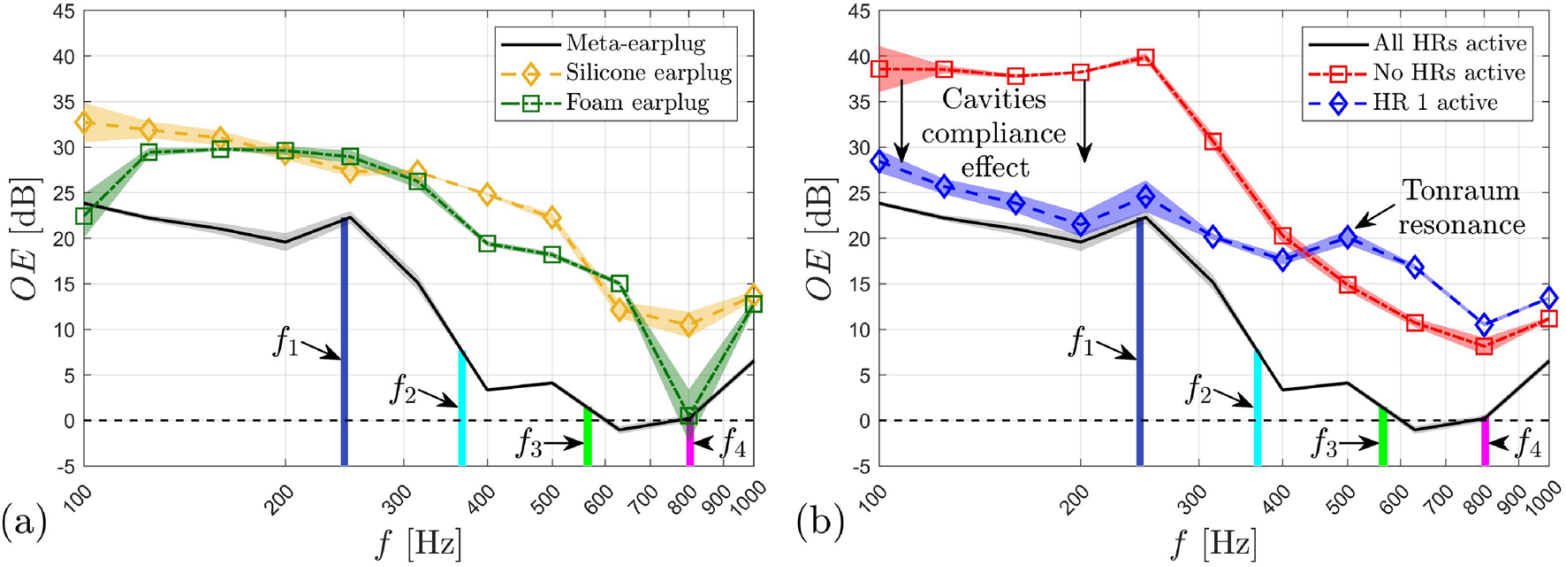
(**a**) Occlusion effect (mean ± standard deviation) induced by the meta-earplug compared to a silicone and a foam earplugs. (**b**) Occlusion effect (mean ± standard deviation) induced by the meta-earplug with all HRs active, no HRs active and only HR#1 active. Results are presented in 3rd octave bands. Vertical coloured lines indicate frequencies of absorption peaks of the meta-earplug, i.e., ${f}_{1}=245$ Hz, ${f}_{2}=367$ Hz, ${f}_{3}=565$ Hz, and ${f}_{4}=803$ Hz. In (**b**), note that the cavities compliance effect only applies to frequencies below 200 Hz.

We now investigate experimentally the benefit of the meta-earplug for reducing the occlusion effect. An associated theoretical investigation is provided in Supplementary Materials. Figure 4a displays the experimental objective occlusion effect (mean ± standard deviation) induced by the meta-earplug inserted in an artificial ear specifically designed for occlusion effect assessment. For this purpose, the artificial ear includes skin, cartilaginous and bony parts surrounding a cylindrical earcanal of length 29 mm and radius 3.75 mm. Note that the treatment of the earcanal as a cylinder with constant radius does not change the basics of bone-conducted acoustic wave propagation^5–7^, at least at low frequencies, compared to a realistic 3D shaped earcanal but greatly simplifies the experimental setup. For further details about the artificial ear used in this work and the associated experimental setup, the reader is referred to “Sample manufacturing and experiments” section. In Fig. 4a, the occlusion effect induced by the proposed meta-earplug is compared to a homemade silicone earplug and a commercial roll-down foam earplug inserted at the same insertion depth, i.e., around 9 mm from the earcanal entrance. Results of the occlusion effect are presented in 3^rd^ octave band in the frequency range 100 Hz–1 kHz. Vertical coloured lines indicate absorption peaks frequencies of the meta-earplug (i.e., ${f}_{1}$–${f}_{4}$).

In agreement with experimental literature data obtained on human subjects^5,26^, the occlusion effect displayed in Fig. 4a for silicone and foam earplugs is shown to decrease with frequency from approximately 30 to 10 dB. This decrease is explained by the change in the character of the acoustic impedance of the earcanal seen by its wall between the mass-controlled open state and the compliance-controlled occluded state^6^. According to Fig. 4a, we can see that the meta-earplug provides a significant broadband reduction of the occlusion effect compared to silicone and foam earplugs. This reduction reaches 15 to 20 dB (depending on which earplug is compared to the meta-earplug) in the 3rd octave band centered at 400 Hz. Above this frequency, the occlusion effect induced by the meta-earplug remains lower than 5 dB. Regarding the silicone and the foam earplugs, their difference in occlusion effect is deemed to come from their difference in Poisson’s ratio^7^, which influences the vibro-acoustic contribution of their medial surface to the sound pressure level generated in the occluded earcanal. For the meta-earplug, the occlusion effect reduction rather comes from the acoustic properties of its medial surface.

In order to examine the acoustic behaviour of the meta-earplug for reducing the occlusion effect, Fig. 4b displays the experimental occlusion effect measured with all HRs active, no HRs active (neck of all HRs are obstructed) and only HR#1 active. When no HRs are active, the meta-earplug medial surface is acoustically rigid and its input acoustic impedance tends to infinity. Compared to the case with no HRs active, the meta-earplug with all HRs active provides a reduction of the occlusion effect from 15 to 5 dB between 100 Hz and 1 kHz and this reduction reaches almost 20 dB at 200 Hz. Between 200 and 900 Hz, this reduction is driven by the quasi-perfect broadband absorption of the meta-earplug medial surface whose input impedance approximately matches the characteristic impedance of air (see Fig. 2a,b). Below 200 Hz, the perfect absorption behaviour of the meta-earplug with all HRs active vanishes (see Fig. 2a) so the reduction of the occlusion effect it provides rather comes from the acoustic compliance of its cavities which decreases its input impedance depending on their total volume (see Supplementary Materials for more detail). This phenomenon is purely reactive and is similar to the reduction of the occlusion effect observed when using large earmuffs^3,5^.

When HR#1 only is active, Fig. 4b shows that the meta-earplug benefits from (i) the resonator volume for reducing the occlusion effect below 200 Hz and (ii) the perfect absorption ensured by its critical coupling at its resonance frequency around 250 Hz (see Fig. 2c). Above this frequency, however, the acoustic absorption of the meta-earplug vanishes. In the 3rd octave band centered at 500 Hz and above, Fig. 4b shows that the occlusion effect induced by HR#1 only is even larger than the occlusion effect produced when no HRs are active. This phenomenon is explained by the Tonraum acoustic resonance^27^ resulting from the coupling of HR#1 to another finite volume, i.e., the earcanal cavity. When all HRs are active, the quasi-perfect broadband absorption completely damps Tonraum resonances that could occur with HR#1, HR#2 and HR#3 and shifts out of the frequency range of interest the Tonraum resonance associated with HR#4 where the occlusion effect is already low (typically above 1 kHz). Theoretical results provided in Supplementary Materials compare well with current experimental data and support the explanation given here regarding the acoustic behaviour of the meta-earplug for reducing the occlusion effect. We also provide an investigation of the influence of the reflection coefficient of the earplug medial surface on the occlusion effect. In addition, the influence of the earcanal radius at the insertion depth of the meta-earplug on the reflection coefficient of this latter is also studied in Supplementary Materials.

Finally, by comparing Fig. 4a,b, we can see that the meta-earplug with all HRs blocked induces a 5 to 10 dB higher occlusion effect compared to silicone and foam earplugs below 250 Hz. This increase in occlusion effect does not come from the acoustic properties of the meta-earplug medial surface, which acts as an acoustically rigid surface when all HRs are blocked, similarly to silicone and foam earplugs. This increase rather originates from the mechanical behaviour of the meta-earplug and its coupling with the earcanal wall and the earcanal cavity.

### Insertion loss

**Figure 5:**
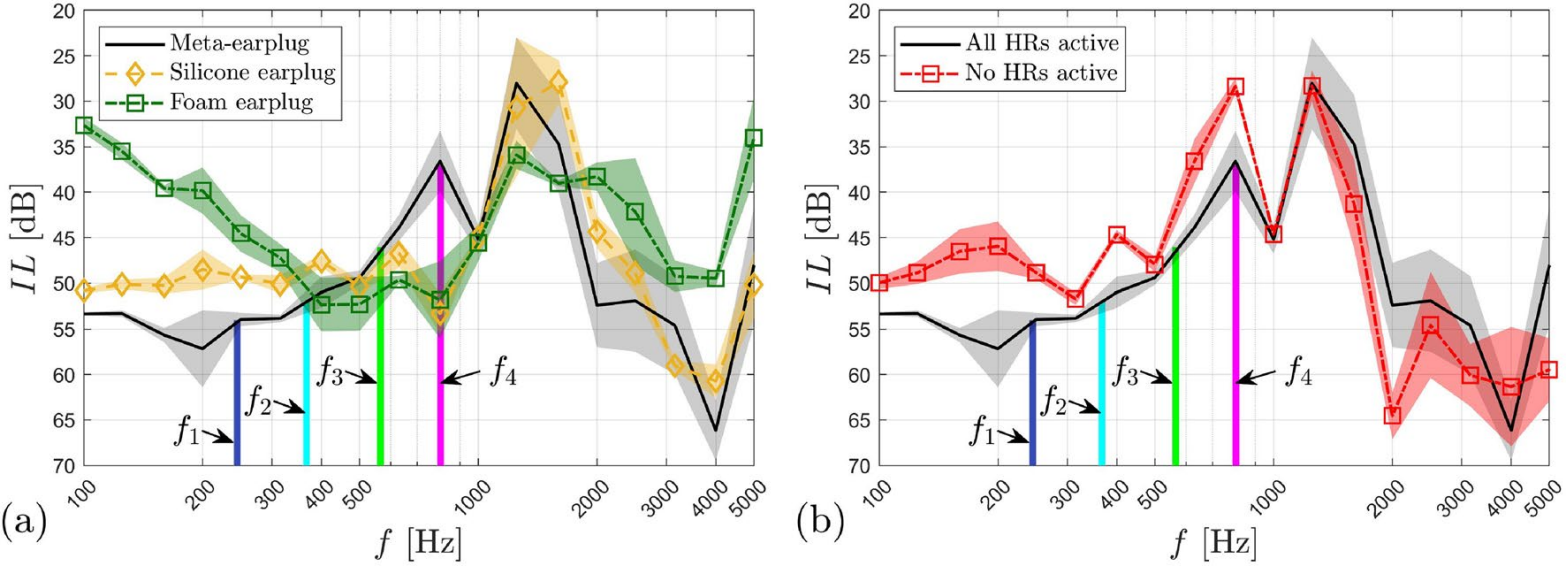
(**a**) Insertion loss (mean ± standard deviation) provided by the meta-earplug compared to a silicone and a foam earplug. (**b**) Insertion loss (mean ± standard deviation) provided by the meta-earplug with all HRs active and no HRs active. Results are presented in 3rd octave bands. Vertical coloured lines indicate frequencies of absorption peaks of the meta-earplug, i.e., ${f}_{1}=245$ Hz, ${f}_{2}=367$ Hz, ${f}_{3}=565$ Hz, and ${f}_{4}=803$ Hz.

The primary function of an earplug being to ensure sound attenuation for users, we finally investigate the insertion loss provided by the meta-earplug using a standard artificial test fixture (see “Sample manufacturing and experiments” section for further details about the experimental setup). Figure 5a displays the experimental insertion loss (mean ± standard deviation) of the meta-earplug compared to that of silicone and foam earplugs inserted at the same insertion depth, i.e., around 9 mm from the earcanal entrance. Insertion loss results are presented in 3rd octave band in the frequency range 100 Hz–5 kHz. Note the reverse direction of the y-axis which presents the insertion loss from the highest (bottom) to the lowest (top) value, as usual in the hearing protection field. Again, vertical coloured lines indicate frequencies of absorption peaks of the meta-earplug (i.e., ${f}_{1}$–${f}_{4}$).

According to Fig. 5a, the meta-earplug provides an insertion loss that has a curve similar to the silicone earplug. The curve of the silicone earplug compares well with literature data^28,29^. The local minimum of the silicone earplug insertion loss that occurs in the frequency band centered at 1.6 kHz comes from a piston-like mode of deformation of the system and depends on the apparent stiffness of the earplug compressed in the earcanal wall skin layer of the artificial test fixture^28^. The insertion loss of the meta-earplug presents 2 local minima in the frequency bands centered at 800 Hz and 1.25 kHz. Both minima might correspond to 2 modes of deformation of the system in different planes since the meta-earplug is not axi-symmetrical with respect to the earcanal axis (see more details in next paragraph). Compared to the foam earplug, the insertion loss provided by the meta-earplug is significantly higher below 400 Hz and above 2 kHz. Between these frequencies, the insertion loss of the meta-earplug drops due to its modes of deformation. Overall, the meta-earplug achieves a suitable insertion loss for hearing protection purpose.

The insertion loss measured on artificial test fixture depends on both (i) the transmission coefficient through the earplug from the surrounding environment to the earcanal, and (ii) the reflection coefficient of the earplug medial surface, which influences the amplitude of the multiple reflections that occur in the occluded earcanal^30^. For silicone and foam earplugs, the reflection coefficient of their medial surface is approximately equal to 1 so the insertion loss they provide only depends on their transmission coefficient. For the meta-earplug, however, the low reflection coefficient of its medial surface has some influence at low frequencies on the insertion loss it provides. To investigate this phenomenon, Fig. 5b displays the insertion loss of the meta-earplug measured with all HRs active and no HRs active (i.e., necks of HRs were not 3D printed). The removal of all HRs is likely to decrease the transmission coefficient of the meta-earplug from the surrounding environment to the earcanal while increase the reflection coefficient of the meta-earplug medial surface. Below 1 kHz, Fig. 5b shows that the insertion loss is reduced (up to 10 dB) when no HRs are active. Hence, the quasi-perfect broadband absorption provided by all HRs active has a significant effect for increasing the insertion loss provided by the meta-earplug at low frequencies. Above 1 kHz, however, the insertion loss of the meta-earplug is generally higher when no HRs are active which is most likely due to the decrease of the transmission coefficient while the reflection coefficient is similar between both cases in this frequency range. Finally, at 800 Hz and 1.25 kHz, local minima of the insertion loss are observed with all HRs active and no HRs active, which supports that these minima originate from mechanical resonances of the meta-earplug/artificial skin system. At 800 Hz, however, the insertion loss is greater when all HRs are active due to the quasi-perfect absorption behaviour of the meta-earplug medial surface.

## Discussion

In this paper, we presented the first meta-earplug capable of reducing the occlusion effect while providing a suitable sound attenuation in a passive way using its own vibro-acoustic behaviour. The reduction of the occlusion effect was ensured by the impedance matching of the meta-earplug medial surface to the characteristic impedance of air. The impedance matching was based on the quasi-perfect broadband absorption behaviour of the meta-earplug medial surface in the frequency range 200–900 Hz, which covers most frequencies where the occlusion effect is noticeable. Hence, we demonstrated here a new solution for passive hearables to reduce the objective occlusion effect without using deep-fitting^8^, which can induce mechanical discomfort, or vent/open-fitting^10,13^, not adapted to hearing protection purpose.

In addition to providing a suitable sound attenuation (similar to that of a silicone earplug), the meta-earplug could improve the sound attenuation of double hearing protectors (DHPs), i.e., the combination of earplugs and earmuffs. When combined, earplugs and earmuffs provide an overall sound attenuation which is less than the sum of their respective sound attenuation taken individually^31^: a phenomenon referred to as the DHP effect. Recently, this phenomenon has been explained by the outer ear structure-borne sound transmission induced by the external acoustic excitation that by-pass the air-borne pathway through the earplug, due to the presence of the earmuff^32,33^. The outer ear structure-borne pathway corresponds to the vibration of the earcanal wall, which is also the source of the occlusion effect. Hence, the low acoustic impedance of the meta-earplug medial surface, which decreases the occlusion effect, could also decrease the DHP effect.

To conclude, the meta-earplug presented here opens several ways for developing next generation of passive earplugs. In future work, the effective efficiency of the meta-earplug for reducing the occlusion effect discomfort should be studied on human subjects. This challenging task will require to adapt the geometry of the meta-earplug to real human outer ear. Also, other types of resonators such as mass-membrane systems or poro-elastic plates could be used for extending the frequency range of quasi-perfect broadband absorption or minimize the size or the mass of the current meta-earplug reducing the occlusion effect. In addition, acoustic properties of the meta-earplug could be optimized for different purposes such as flat broadband sound attenuation or selective frequency band of the sound attenuation.

## Methods

### Models of the meta-earplug medial surface impedance

#### Theoretical model

In this section, we detail the theoretical model developed for the optimization process of the reflection coefficient of the meta-earplug medial surface. The temporal dependency is taken as ${e}^{j\omega t}$. Under the assumption of normal incidence plane wave propagation, the reflection coefficient is defined by

1 $$R=\frac{Z-{Z}_{0}}{Z+{Z}_{0}},$$

where ${Z}_{0}$ is the air characteristic impedance and $Z$ is the input acoustic impedance of the meta-earplug including the 4 HRs in parallel and given by

2 $$Z={\left(\sum_{n=1}^{4}\frac{1}{{Z}_{HR}^{[n]}}\right)}^{-1}.$$

The TMM is used to model the input impedance ${Z}_{HR}$ of each HR composing the meta-earplug. Geometrical parameters used in the following depend on each HR. The transfer matrix $\mathbf{T}$ relates the acoustic pressure $p$ and the particle velocity $v$ (in the longitudinal direction along the ${x}_{1}$-axis) from the neck entrance (${x}_{1}=0$, see Fig. 1d) to the back of the cavity (${x}_{1}=l+L$) such that

3 $${\left[\begin{array}{l}p\\ v\end{array}\right]}_{entrance}=\mathbf{T}{\left[\begin{array}{l}p\\ v\end{array}\right]}_{back}=\left[\begin{array}{ll}{T}_{11}& {T}_{12}\\ {T}_{21}& {T}_{22}\end{array}\right]{\left[\begin{array}{l}p\\ v\end{array}\right]}_{back}.$$

Since HRs are rigidly backed (i.e., ${\left.\widehat{v}\right|}_{back}=0$), the input impedance ${Z}_{HR}$ of each one is obtained by

4 $${Z}_{HR}=\frac{{T}_{11}}{{T}_{21}}.$$

The transfer matrix $\mathbf{T}$ of each HR is defined by the product of the transfer matrices of (i) the change in section at ${x}_{1}=0$ between the earcanal and the neck (${\mathbf{T}}_{up}$), (ii) the neck itself (${\mathbf{T}}_{neck}$), (iii) the change in section at ${x}_{1}=l$ between the neck and the cavity (${\mathbf{T}}_{down}$), (iv) the portion of cavity which is not filled with foam (${\mathbf{T}}_{cav}$), and finally (v) the foam layer (${\mathbf{T}}_{foam}$) such that

5 $$\mathbf{T}={\mathbf{T}}_{up}{\mathbf{T}}_{neck}{\mathbf{T}}_{down}{\mathbf{T}}_{cav}{\mathbf{T}}_{foam}.$$

Transfer matrices of change in section (${\mathbf{T}}_{up}$ and ${\mathbf{T}}_{down}$) account for the continuity of acoustic pressure and volume flow across the interface and are defined as follows

6 $${\mathbf{T}}_{up}=\left[\begin{array}{ll}1& 0\\ 0& {S}_{neck}/{S}_{EC}\end{array}\right],$$

7 $${\mathbf{T}}_{down}=\left[\begin{array}{ll}1& 0\\ 0& {S}_{cav}/{S}_{neck}\end{array}\right],$$

where ${S}_{EC}$ is the earcanal cross-section area at the meta-earplug insertion depth and ${S}_{neck}=\pi {\left({r}_{neck}\right)}^{2}$ and ${S}_{cav}={e}_{cav}{h}_{cav}$ are cross-section areas of neck and cavity respectively.

Transfer matrices of necks, cavities and foam layers (${\mathbf{T}}_{neck}$, ${\mathbf{T}}_{cav}$, and ${\mathbf{T}}_{foam}$) are similarly defined by

8 $${\mathbf{T}}_{i}=\left[\begin{array}{ll}\mathrm{cos}({k}_{eq,i}{l}_{i})& j{Z}_{eq,i}\mathrm{sin}({k}_{eq,i}{l}_{i})\\ \frac{-1}{j{Z}_{eq,i}}\mathrm{sin}({k}_{eq,i}{l}_{i})& \mathrm{cos}({k}_{eq,i}{l}_{i})\end{array}\right],$$

where $i\in \left\{neck ; cavity ; foam\right\}$, $j$ is the imaginary number, ${l}_{i}$ is the length of either neck, portion of cavity without foam or foam layer while ${Z}_{eq,i}$ and ${k}_{eq,i}$ are the equivalent impedance and wavenumber accounting for visco-thermal effects. In the case of neck, effective length ${l}_{eff,neck}={l}_{neck}+{l}_{up}+{l}_{down}$ is used instead of geometrical length ${l}_{neck}$ to account for lengths correction ${l}_{up}$ and ${l}_{down}$ respectively due to acoustic radiation at the discontinuity from the neck to the earcanal and due to pressure radiation at the discontinuity from the neck to the HR cavity. Here, these lengths correction are well approximated by^34^

9 $${l}_{up}=\frac{8{r}_{neck}}{3\pi }\left(1-1.25\frac{{S}_{neck}}{{S}_{EC}}\right),$$

10 $${l}_{down}=\frac{8{r}_{neck}}{3\pi }\left(1-1.25\frac{{S}_{neck}}{{S}_{cav}}\right).$$

For necks and portion of cavities without foam, ${Z}_{eq,i}$ and ${k}_{eq,i}$ are defined using a low reduced frequency model^35^. For foam layers, ${Z}_{eq,foam}$ and ${k}_{eq,foam}$ used in Eq. (8) are computed using the Johnson–Champoux–Allard equivalent fluid model^36^. Tables 2 and 3 respectively summarizes the properties of air at normal room conditions and the macroscopic properties of the melamine foam.

**Table 2:** Properties of air at normal room conditions ($T=22 ^\circ$C, ${P}_{atm}=101,300$ Pa and $HR=45\mathrm{\%}$): air density ${\rho }_{0}$, sound speed ${c}_{0}$, dynamic viscosity $\eta$, specific heats ratio $\gamma$, heat capacity at constant pressure ${C}_{p}$, thermal conductivity coefficient $\kappa$ and Prandtl number ${P}_{r}$.

${\rho }_{0}$[kg/m^3^]	${c}_{0}$[m/s]	$\eta$[Pa s]	$\gamma$[1]	${\mathrm{C}}_{p}$[J/kg/K]	$\kappa$[W/m/K]	${P}_{r}$[1]
$1.19$	$345.5$	$1.83\times 1{0}^{-5}$	$1.4$	$1.002\times 1{0}^{3}$	$0.025$	$0.707$

**Table 3:** Macroscopic properties of the melamine foam: porosity $\phi$, tortuosity ${\alpha }_{\infty }$ (high frequency limit), air flow resistivity $\sigma$, viscous and thermal characteristic lengths $\Lambda$ and $\Lambda{^{\prime}}$.

$\phi$[1]	${\alpha }_{\infty }$[1]	$\sigma$[N m^−4^ s]	$\Lambda$[m]	$\Lambda{^{\prime}}$[m]
$0.971$	$1$	$7566$	$8.7\times 1{0}^{-5}$	$1.63\times 1{0}^{-4}$

#### Numerical model

In order to verify the semi-analytical model, we developed a numerical model of the meta-earplug based on the FEM using COMSOL Multiphysics^®^ 5.6. In this purely acoustical model (the structure of the meta-earplug is considered rigid and motionless), the acoustic pressure satisfies Helmholtz equation at all points of the fluids. Visco-thermal losses in necks and portion of cavities without foam are accounted for using the low reduced frequency model^35^ adapted for circular and rectangular sections. Foam layers are taken into account as equivalent fluid using the Jonhson–Champoux–Allard model^36^. Compared to the theoretical approach, the numerical model naturally accounts for (i) the exact acoustic radiation of necks in both the main waveguide and the cavities and (ii) the direct interaction of HRs due to the evanescent coupling. However, this has little effect on the results and both approaches compare well (see “Acoustic properties of the meta-earplug medial surface” section). The geometry of the numerical model was meshed according to a criterion of at least six 10-noded (quadratic) tetrahedral elements per wavelength at 1 kHz (maximum frequency of interest) in order to achieve convergence.

### Sample manufacturing and experiments

**Figure 6:**
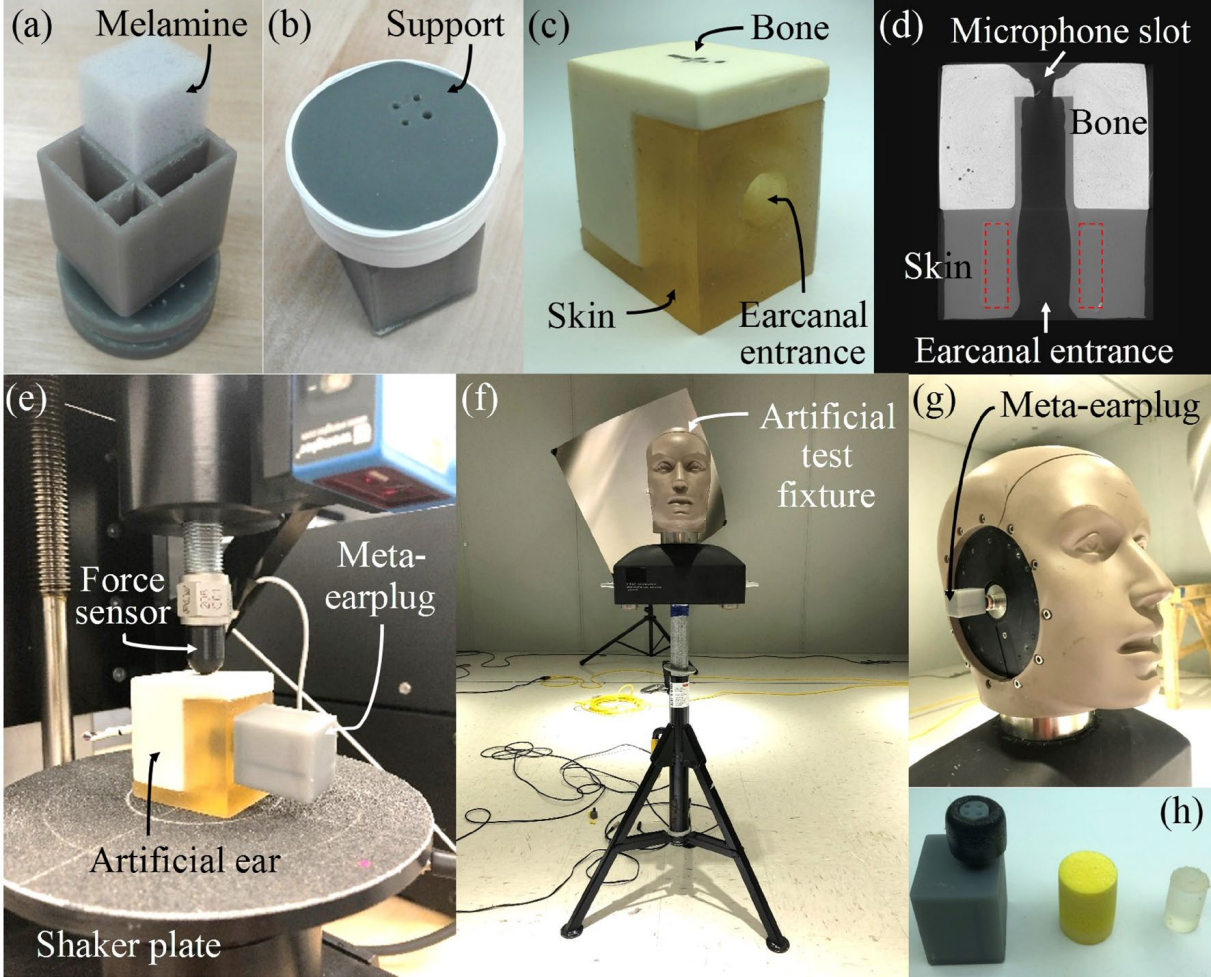
(**a**) Lateral and (**b**) medial view of the meta-earplug including a built-in support for impedance sensor measurement and showing the insertion of melamine foam in HR cavity before gluing the back-plate. (**c**) Artificial ear designed for occlusion effect measurements and (**d**) CT-scan performed in the horizontal plane showing the geometry of the artificial earcanal and its surrounding tissues (red dotted rectangles indicate the position of the cartilaginous ring surrounding the earcanal but difficult to distinguish from the skin tissue). (**e**) Experimental setup for occlusion effect assessment using a QMA facility. (**f**) Experimental setup for measuring the insertion loss of earplugs using an artificial test fixture placed in a reverberant room. (**g**) Meta-earplug inserted in the earcanal of the artificial test fixture. (**h**) Size comparison between the meta-earplug (left), the foam (middle) and the silicone (right) earplugs used in the current work.

Samples of the meta-earplug were 3D printed based on stereo-lithography technique using a photosensitive epoxy polymer (Grey pro V1 resin, Form 2 printer, Formlabs^®^, MA, USA). The mechanical properties of the post-cured resin make the 3D printed structures acoustically rigid (2.6 GPa Young’s modulus and 1200 kg/m^3^ density). Foam layers were cut manually from melamine panel and inserted in each HR (see Fig. 6a). Finally, a 3D printed back-plate was glued to close the cavities of the meta-earplug using cyanoacrylate adhesive.

Acoustic properties of the meta-earplug medial surface were assessed using a 10 mm diameter impedance sensor^25^. For this purpose, the sample was built with an integrated support adapted for the impedance measurement (see Fig. 6b). Hence, the eartip of the meta-earplug was not included to correspond to the assumptions of the theoretical model used in the optimisation process. From the measured acoustic impedance ${Z}_{samp}$ of the sample including the integrated support, the acoustic impedance $Z$ of the meta-earplug medial surface of area ${S}_{EC}$ is obtain by

11 $$Z={Z}_{samp}\frac{{S}_{EC}}{{S}_{IS}},$$

where ${S}_{IS}$ is the impedance sensor cross-section area. Then, the measured reflexion coefficient is computed using Eq. (1).

The occlusion effect induced by the meta-earplug was assessed at normal room temperature using a dedicated artificial ear including the earcanal and its surrounding tissues (i.e., soft, cartilaginous and bony tissues). This artificial ear is displayed in Fig. 6c and was designed in the ICAR (“Infrastructure Commune en Acoustique pour la Recherche”) laboratory at ETS for bone-conducted stimulation^37^. The geometry of the present artificial ear is a simplification of the 3D geometry of a human outer ear constructed from magnetic resonance images of a human subject in Ref.^38^. In the simplification process, the earcanal was considered as cylindrical and volumes of the earcanal surrounding tissues were kept equivalent to those in the original 3D geometry. Figure 6d displays a computed tomography scan of the artificial ear in the horizontal plane. The transition between soft tissue (denoted as skin) and bony tissue approximately occurs at the half-length of the earcanal, in agreement with literature data^5^. Due to inherent manufacturing difficulties, the geometry of the earcanal is not exactly cylindrical in the cartilaginous region (see the red dotted rectangles in Fig. 6d). The artificial ear was manufactured by True Phantom solutions (Windsor, Canada) based on the provided CAD model. As in Ref.^38^, materials used for the artificial ear were a polyurethane based material (35 shore 00) for the soft tissues (i.e., skin), a polyurethane based material with a higher stiffness (65 shore A) for the cartilage, and an epoxy based ceramic material for the bony part. The bone-conducted stimulation of the artificial ear was ensured by the shaker of a quasi-static mechanical analyser (Mecanum, Canada) referred to as a QMA. The artificial ear was slightly compressed (1%) between the shaker plate and the force sensor (see Fig. 6e). This setup dictated the choice of a parallelepipedic geometry for the artificial ear. The acoustic pressure was measured using an electret microphone inserted in a hole located in the bony region of the artificial ear lateral surface (see Fig. 6d). Hence, the acoustic effect of the eardrum was not accounted for but rather simplified to an acoustically rigid surface which tends to slightly overestimate the resulting occlusion effect by a few dBs. Transfer functions ${H}_{BC}^{occl}$ and ${H}_{BC}^{open}$ defined between the acoustic pressure at the microphone and an accelerometer placed on the shaker plate were measured respectively in open and occluded earcanals. The occlusion effect was then computed by

12 $$OE=20{\mathrm{log}}_{10}\left(\left|{H}_{BC}^{occl}/{H}_{BC}^{open}\right|\right).$$

The insertion loss provided by the meta-earplug was characterized at normal room temperature using a commercial artificial test fixture (G.R.A.S. 45CB, G.R.A.S. Sound & Vibration AS, Denmark) placed in a reverberant chamber (see Fig. 6f), based on American standard^39^. The ear simulator is composed of a cylindrical earcanal of radius equal to 3.75 mm and partially covered by a 10 mm long silicon layer (from the earcanal entrance) and terminated by an IEC 60318-4 coupler simulating the tympanic impedance. The pinna simulator was removed because the geometry of the meta-earplug was not adapted to fit into it (see Fig. 6g). A pink noise of approximately 110 dB overall was generated by 4 speakers (type JAMO^®^ S628, Klipsch Group, Inc., Denmark) in the reverberant room. Sound pressure levels ${L}_{p,AC}^{open}$ and ${L}_{p,AC}^{occl}$ induced by air-conducted (subscript AC) stimulation were measured respectively in open and occluded earcanals using the microphone included in the ear simulator (G.R.A.S. 40AG, G.R.A.S. Sound & Vibration AS, Denmark). The insertion loss was then computed by

13 $$IL={L}_{p,AC}^{open}-{L}_{p,AC}^{occl}.$$

Occlusion effect and insertion loss of silicone and foam earplugs (see Fig. 6h) were also measured using the aforementioned setups for comparison purpose with the meta-earplug performance. The silicone earplug was 3D printed using a flexible resin (Elastic 50A, Formlabs^®^, MA, USA) corresponding to a 50A Shore durometer material with a quasi-incompressible mechanical behaviour. Its diameter was chosen slightly larger than the earcanal itself to adequately seal the earcanal entrance. The foam earplug was a 3 M E.A.R Classic^®^ earplug. For each meta-earplug (including all HRs active, no HRs active and HR#1 active) and earplug (silicone and foam materials), the insertion depth was approximately equal to 9 mm for both occlusion effect and insertion loss measurements. Also, measurements were repeated 3 times (earplug inserted and removed each time) for all earplugs to assess the variability associated with mounting conditions in both experimental setups. In addition, sound pressure level measured in the artificial ear and the artificial test fixture were always checked to be at least 10 dB higher than the background noise.

## Supplementary Information

Supplementary Information.

## Data Availability

The datasets generated during and/or analysed during the current study are available from the corresponding author on reasonable request.
