# Supplementary material for: Reduction of the occlusion effect induced by earplugs using quasi perfect broadband absorption
Source: Sci Rep. 2022 Sep 12;12:15336. doi: 10.1038/s41598-022-19641-3 (PMC9468357; doi:10.1038/s41598-022-19641-3)
Supplement: Supplementary file 1 — Supplementary Information. [file 41598_2022_19641_MOESM1_ESM.pdf]

SUPPLEMENTARY MATERIALS:
Reduction of the occlusion effect induced by earplugs using quasi perfect broadband absorption

Kévin Carillo[1], Franck Sgard[2], Olivier dazel[3] and Olivier Doutres[1]

[1]École de technologie supérieure (ETS), Montréal, Québec, Canada
[2]Institut de recherche Rober-Sauvé en santé et en sécurité du travail (IRSST), Montréal, Québec, Canada
[3]Laboratoire d'Acoustique de l'Université du Mans (LAUM), UMR 6613, Institut d'Acoustique – Graduate School (IA-GS), CNRS, Le Mans Université, France

I. ABSORPTION BANDWIDTH OF ISOLATED HELMHOLTZ RESONATORS

This section describes how the absorption bandwidth of isolated HRs can be approximated from the representation of their reflection coefficient in the complex frequency plane. The absorption bandwidth $\Delta f_{[n]}$ of the nth isolated HR is related to its quality factor $Q_{[n]}$ by $\Delta f_{[n]} = f_{[n]}/Q_{[n]}$ with $f_{[n]}$ the resonance frequency of the HR. In addition, the quality factor can be decomposed such as[1] $Q^{-1} = Q_{leak}^{-1} + Q_{loss}^{-1}$ where Q_{leak} and Q_{loss} are the quality factors associated with the leakage of the resonator in the waveguide and with the losses in the HR, respectively. Q_{leak} can be calculated in the lossless case from the complex frequency plane by[2] $Q_{leak} = 0.5\Re\left(f^p\right)/\Im\left(f^p - f^z\right)$ with f^p and f^z the complex frequency of pole and zero respectively. Since the main influence of losses is a vertical shift of pairs of pole and zero in the complex frequency plane, the previous equation is approximately valid in the lossy case. On the other hand, Q_{loss} depends on the acoustic energy dissipation of each HR. When the critical coupling condition is fulfilled, then $Q_{leak} = Q_{loss}$. Hence, $Q = Q_{leak}/2$ and the absorption bandwidth can be approximated by $\Delta f_{[n]} = 2\Im\left(f_{[n]}^p - f_{[n]}^z\right)$.

In Fig. S1(a-d), we display the acoustic absorption α of each isolated HR computed using the TMM approach. Bandwidths $\Delta f_{[n]}$, with $n \in \{1;2;3;4\}$ the index of each HR, are indicated in continuous blue lines and are computed from the intersection of absorption curves with horizontal dashed red lines

at -3 dB from absorption peaks. The resulting bandwidths are summarised in Table S1 and we can see that those of HR#3 and HR#4 are basically the same. Indeed, since the distance between pole and zero is the same for HR#3 and HR#4 (determined by the change in section between the waveguide and their neck of equal radius), their absorption bandwidths are also approximately equal, at least when they are isolated. Finally, we can see from Table S1 that the computation of absorption bandwidth from the complex frequency plane gives similar results compared to the graphic-reading method.

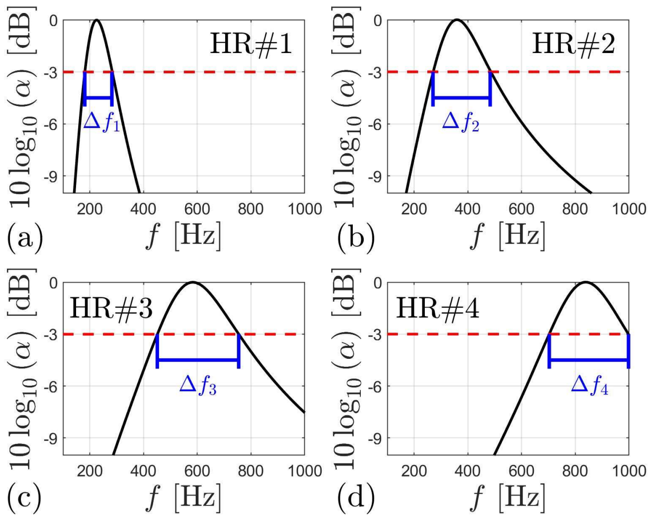

FIG S1: (a-d) Acoustic absorption α of each isolated HR computed using the TMM approach. Bandwidths $\Delta f_{[n]}$, with $n \in \{1;2;3;4\}$ the index of each HR, are indicated in continuous blue lines and are computed from the intersection of absorption curves with horizontal dashed red lines at -3 dB from absorption maxima.

	$\Delta f_{[n]}$ (calculated from Fig. S1)	$\Delta f_{[n]} \approx 2\Im\left(f_{[n]}^{p} - f_{[n]}^{z}\right)$
HR#1	161 Hz	144 Hz
HR#2	214 Hz	210 Hz
HR#3	303 Hz	292 Hz
HR#4	295 Hz	294 Hz

Table S1: Acoustic absorption bandwidth $\Delta f_{[n]}$, with $n \in \{1 ; 2 ; 3 ; 4\}$, of each isolated HR calculated from Fig. S1 at -3 dB from absorption peaks and computed from the representation of the reflection coefficient in the complex frequency plane through the distance between pole and associated zero of isolated HRs.

II. INFLUENCE OF THE EARCANAL CROSS-SECTION ON THE META-EARPLUG REFLECTION COEFFICIENT

The quasi-perfect broadband absorption of the meta-earplug results from the balance between the energy leakage from the meta-earplug to the coupling duct (i.e., the earcanal) and the energy dissipation inside the meta-earplug. This balance depends, among other parameters, on the change in section, at the meta-earplug medial surface, between the earcanal and each HR included in the meta-earplug. The current meta-earplug was optimized for an earcanal cross-section equivalent to a circular area of radius 3.75 mm. In practice, the earcanal cross-section to which the meta-earplug would be coupled could differ from that used in the optimization process due to the variety of earcanal size among individuals. Hence, we wonder if the current meta-earplug could be used for different earcanal cross-section than the one for which the meta-earplug was optimized.

Figure S2 displays the reflection coefficient of the meta-earplug of medial surface $S_{EC} = \pi r_{EC}^2$ as a function of both the radius r_{EC} of the earcanal and the frequency, computed from (a) the TMM approach and (b) the experimental data. Black continuous lines represent the isoline $|R|^2 = 0.1$. Theoretical and experimental results are in good agreement. We can see that the reflection coefficient of the meta-earplug generally increases with the radius of its medial surface because the balance between the energy leakage and the energy dissipation is broken. As a consequence, the resulting occlusion effect would also increase, as later shown in Fig. S4(d). However, when the radius of the medial surface decreases, its reflection coefficient is less influenced (see Fig. S2(a-b)).

To investigate this phenomenon, Fig. S2(c) displays the trajectories of poles (dotted colour-lines) and zeros (solid colour-lines) of isolated HRs of the meta-earplug computed in the complex frequency plane as a function of the earcanal radius r_{EC} using the TMM approach. Diamond and circle symbols represent poles and zeros of isolated HRs when $r_{EC} = 3.75$ mm, i.e., the earcanal radius used in the optimization process. When the radius of the earcanal increases from the initial value of $r_{EC} = 3.75$ mm, we can see that the zeros are shifted above the real frequency axis due to an excess of energy dissipation compared to the energy leakage and the perfect absorption of each HR vanishes. In addition, the distance between poles and their associated zeros decreases because the change in section between each neck and the earcanal increases. In consequence, the absorption peak of each HR becomes narrower. On the contrary, when the earcanal radius decreases from the initial value of $r_{EC} = 3.75$ mm, the distance between poles and their associated zeros increases, which broadens the

absorption peaks and contributes to maintaining quasi-perfect broadband absorption even though the critical coupling of each resonator is not achieved (zeros are not on the real frequency axis).

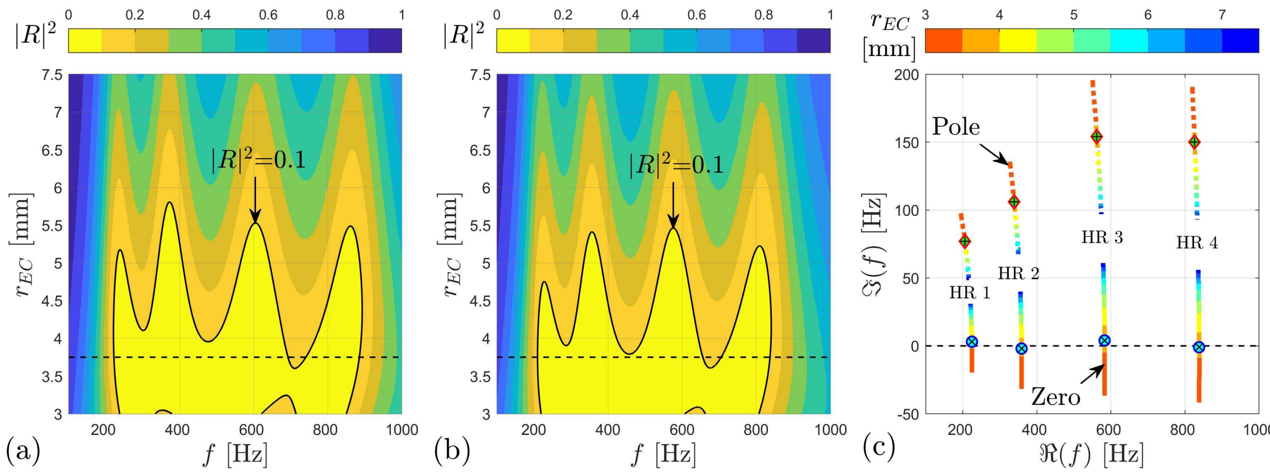

FIG S2: Reflection coefficient of the meta-earplug of medial surface $S_{EC} = \pi r_{EC}^2$ as a function of both the radius r_{EC} of the earcanal and the frequency, computed from (a) the TMM approach and (b) the experimental data. Black continuous lines represent the isoline $|R|^2 = 0.1$. The horizontal dashed line corresponds to the initial earcanal radius $r_{EC} = 3.75$ mm. (c) Trajectories of poles (dotted colour-lines) and zeros (solid colour-lines) of isolated HRs of the meta-earplug computed in the complex frequency plane as a function of the earcanal radius r_{EC} using the TMM approach. Diamond and circle symbols represent poles and zeros of isolated HRs when $r_{EC} = 3.75$ mm, i.e., the earcanal radius used in the optimization process.

III. INFLUENCE OF THE MELAMINE FOAM ON THE META-EARPLUG REFLECTION COEFFICIENT

This section examines the influence of the melamine foam layer in the cavities of the meta-earplug on the reflection coefficient of its medial surface. For this purpose, Fig. S3(a) displays the reflection coefficient of the meta-earplug computed using the TMM with (continuous black curve) and without (dashed red curve) the melamine foam layers. Vertical coloured lines indicate the frequencies of absorption peaks of the meta-earplug (with melamine foam), i.e., $f_1 = 245$ Hz, $f_2 = 367$ Hz, $f_3 = 565$ Hz, and $f_4 = 803$ Hz. We can see two consequences of the removal of the melamine foam from the cavities of the meta-earplug. First, as it is expected, the quasi-perfect broadband absorption vanishes. Second, the minima of the reflection coefficient are shifted towards higher frequencies.

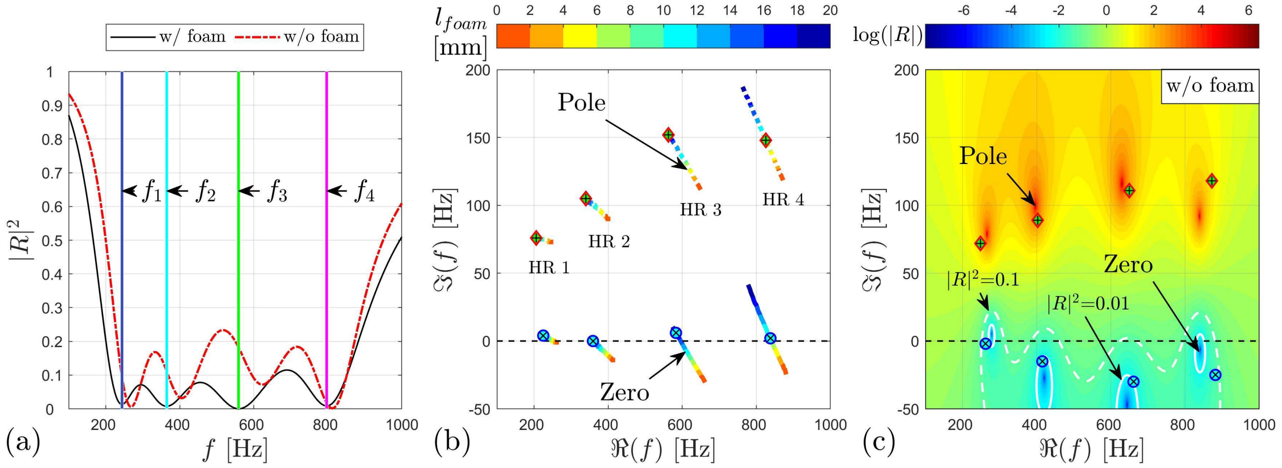

FIG S3: Reflection coefficient of the meta-earplug of medial surface S_{EC} calculated analytically (TMM) with and without melamine foam layer in the cavity of HRs. Vertical coloured lines indicate frequencies of absorption peaks of the meta-earplug including foam layers, i.e., $f_1 = 245$ Hz, $f_2 = 367$ Hz, $f_3 = 565$ Hz, and $f_4 = 803$ Hz. (b) Trajectories of poles (dotted colour-lines) and zeros (solid colour-lines) of isolated HRs of the meta-earplug computed in the complex frequency plane as a function of the melamine foam layer thickness l_{foam} using the TMM approach. Diamond and circle symbols represent poles and zeros of isolated HRs resulting from the optimisation process. (c) Reflection coefficient of the meta-earplug (without melamine foam) in the complex frequency plane analytically computed using the TMM. White continuous (respectively dashed) lines show the isoline $|R|^2 = 0.01$ (respectively $|R|^2 = 0.1$). Diamond and circle symbols represent poles and zeros of HRs isolated from each other (i.e., taken individually). Symbols $\Re$ and $\Im$ represent real and imaginary part respectively.

To explain these phenomena, we display in Fig. S3(b) the trajectories of poles (dotted colour-lines) and zeros (solid colour-lines) of isolated HRs of the meta-earplug in the complex frequency plane as a function of the melamine foam layer thickness l_{foam} using the TMM approach. When the foam layer thickness decreases, pairs of pole and zero of isolated HRs are shifted in both directions of the complex frequency plane. On the one hand, the vertical shift is purely resistive and related to the decrease of losses in the system as the foam layer thickness is reduced. When there is no foam (i.e., $l_{foam} = 0$), however, pole and zero of each pair are not located symmetrically with respect to the real frequencies axis due to the losses that still occur in necks and cavities. On the other hand, the horizontal shift is purely reactive and associated with a decrease of the acoustic compliance of the cavity of HRs when the foam thickness is reduced. Hence, for a given cavity volume, the foam allows for reaching the perfect absorption behaviour at lower frequencies.

Finally, despite the removal of the foam, the critical coupling condition still holds for HR#1 and HR#4 (i.e., the reflection coefficient is approximately zero at these frequencies, see Fig. S3(a)). In the case of HR#1, we can see in Fig. S3(b) that the trajectory of its zero is almost horizontal, hence the critical coupling condition is approximately fulfilled for any thickness of the foam layer. In the case of HR#4, we can see in Fig. S3(b) that it is far from achieving the critical coupling condition when isolated from other HRs. Therefore, the critical coupling of HR#4 rather comes from the indirect interaction between HRs through the waveguide. To visualize this interaction, Fig. S3(c) displays the reflection coefficient of the meta-earplug (without melamine foam) in the complex frequency plane analytically computed using the TMM. Diamond and circle symbols represent poles and zeros of HRs isolated from each other. We can see that the position of poles and zeros in the complex frequency plane are slightly changed from coupled HRs to isolated HRs. For HR#4 in particular, the indirect coupling between HRs shifted its zero almost on the real frequency axis.

IV. THEORETICAL INVESTIGATION OF THE OCCLUSION EFFECT INDUCED BY THE META-EARPLUG

This section presents a simple electro-acoustic model of the occlusion effect in order to provide a theoretical basis to interpret the effect of the meta-earplug for reducing the phenomenon. In this model, the geometry of the earcanal is taken from the artificial ear used in experimental measurement of the occlusion effect. Hence, the earcanal is considered as a cylinder of length $l_{EC} = 29\,\mathrm{mm}$ and radius $r_{EC} = 3.75\,\mathrm{mm}$. Schematics of the earcanal geometry and the associated electro-acoustic model in open and occluded configurations are provided in Fig. S1(a-c).

The vibration of the earcanal wall induced by the bone-conducted stimulation is accounted for as an ideal source Q of volume velocity. When the earcanal is not obstructed (open case), the acoustic impedance seen by the source is approximated at low frequencies by[3]

$$Z_{EC}^{open} = j\omega\left(L_{EC}^{open} + L_{rad}^{open}\right), \tag{S1}$$

where $L_{EC}^{open} = \rho_0 l_c / S_{EC}$ is the acoustic mass of the earcanal defined between the earcanal entrance and the centroid position l_c of the earcanal wall normal velocity and $L_{rad}^{open} = 8\rho_0 / (3\pi^2 r_{EC})$ is the acoustic mass of radiation of the earcanal entrance in the surrounding environment.

When the earcanal is occluded, the contribution of the earplug medial surface in terms of volume velocity is not of primary importance at shallow insertion[4] and can be neglected for the sake of simplicity of the current model. For high enough input impedance Z_{EP} of the medial earplug surface

(i.e., $Z_{EP} \geq Z_0$), the acoustic impedance seen by the earcanal wall is approximated at low frequencies by

$$Z_{EC}^{occl} = \frac{\left(Z_{EP}/S_{EC}\right)\left[j\omega C_{EC}^{occl}\right]^{-1}}{\left(Z_{EP}/S_{EC}\right)+\left[j\omega C_{EC}^{occl}\right]^{-1}}, \tag{S2}$$

where $C_{EC}^{occl} = (l_{EC} - l_{ID})S_{EC}/(\rho_0 c_0^2)$ is the acoustic compliance of the occluded earcanal volume. Note that the influence of the middle ear is not accounted for in the model and the tympanic membrane is rather considered as an acoustically rigid surface, like in the experimental setup. At low frequencies, the influence of the middle ear is mainly governed by the acoustic compliance of the tympanic cavity volume[3]. Neglecting the middle ear compliance slightly increases the acoustic impedance of the occluded earcanal seen by its wall, and therefore, slightly increases the occlusion effect. However, this approximation has little influence on the conclusions drawn here regarding the vibro-acoustic behaviour of the meta-earplug for reducing the occlusion effect.

Finally, acoustic pressure at the tympanic membrane are given by $p_{TM}^{open} = Z_{EC}^{open} q_{EC}^{open}$ and $p_{TM}^{occl} = Z_{EC}^{occl} q_{EC}^{occl}$ where q_{EC}^{open} and q_{EC}^{occl} are the volume velocities imposed in open and occluded case by the earcanal wall. Hence, the occlusion effect is given by

$$OE = 20\log_{10}\left(\frac{Z_{EC}^{occl}}{Z_{EC}^{open}}\Psi_q\right), \tag{S3}$$

where $\Psi_q = q_{EC}^{occl}/q_{EC}^{open}$. The difference in volume velocity between open and occluded cases depends on the insertion depth of the occlusion device. Here, the ratio of volume velocity was adjusted to $\Psi_q = 1/10$ based on comparison between simulated and measured occlusion effect presented in the corresponding paper.

Figure S4(d) displays the occlusion effect induced by the meta-earplug when all HRs are active, no HRs are active and when HR#1 only is active computed using the electro-acoustic model. Vertical coloured lines indicate frequencies of absorption peaks of the meta-earplug (with all HRs active), i.e., $f_1 = 245$ Hz, $f_2 = 367$ Hz, $f_3 = 565$ Hz, and $f_4 = 803$ Hz while the colour-map shows the dependence of the simulated occlusion effect on the reflection coefficient of the earplug medial surface. When no HRs are active, the meta-earplug medial surface is acoustically rigid and its input impedance tends to infinity. Compared to the case with no HRs active, the meta-earplug with all HRs active provides a reduction of the occlusion effect from 20 to 5 dB between 100 Hz and 1 kHz. In the quasi-perfect

broadband absorption regime of the meta-earplug (i.e., between 200 and 900 Hz), the occlusion effect is mainly driven by the reflection coefficient of the meta-earplug medial surface. We can see however that local minima of the occlusion effect do not occur at absorption peaks of the meta-earplug but rather at the resonance frequencies of HRs of the meta-earplug. At these frequencies, the occlusion effect is even lower than the occlusion effect resulting from a zero reflection medial surface because the acoustic impedance of the meta-earplug medial surface is lower than that of the air while a zero reflection medial surface provides an impedance matching with that of the air. Between absorption peak frequencies, it is noteworthy that local maxima of the occlusion effect correspond to local maxima of the reflection coefficient of the meta-earplug medial surface. In this regard, colour-map of Fig. S4(d) shows that the reflection coefficient of the meta-earplug medial surface should not exceed the value of 0.1 for the meta-earplug to significantly reduce the occlusion effect. Hence, to maximise the occlusion effect reduction provided by the meta-earplug, the reflection coefficient of its medial surface must be optimised for several earcanal cross-sections covering the range of variation that exists among individuals. Below 200 Hz, the perfect absorption behaviour of the meta-earplug medial surface vanishes. The occlusion effect provided by the meta-earplug is not governed by its resistive behaviour (which tends to zero) but rather by its reactive behaviour governed by the acoustic compliance of its cavities which decreases the input impedance of the meta-earplug medial surface and the resulting occlusion effect (see Fig. S4(d)).

According to Fig. S4(d), when HR#1 only is active, the occlusion effect is significantly reduced below 200 Hz due to the acoustic compliance added by the resonator volume. At the resonance frequency of HR#1 (around 220 Hz), the critically coupled resonator achieves perfect absorption and the resulting occlusion effect reaches a local minimum. Above this frequency, Fig. S4(d) shows that the occlusion effect of the meta-earplug with only HR#1 active reaches a local maximum at 500 Hz which is even higher than the occlusion effect induced by an infinite impedance. Indeed, in this frequency range, the acoustic absorption of the meta-earplug vanishes and the meta-earplug acoustic impedance is mainly reactive. Hence, the coupled resonance known as the Tonraum resonance[5] can occur between the Helmholtz resonator and the earcanal which acts as a second finite volume for the resonating system. When all HRs are active, Tonraum resonances which occur in the quasi-perfect broadband absorption frequency region of the meta-earplug are completely damped.

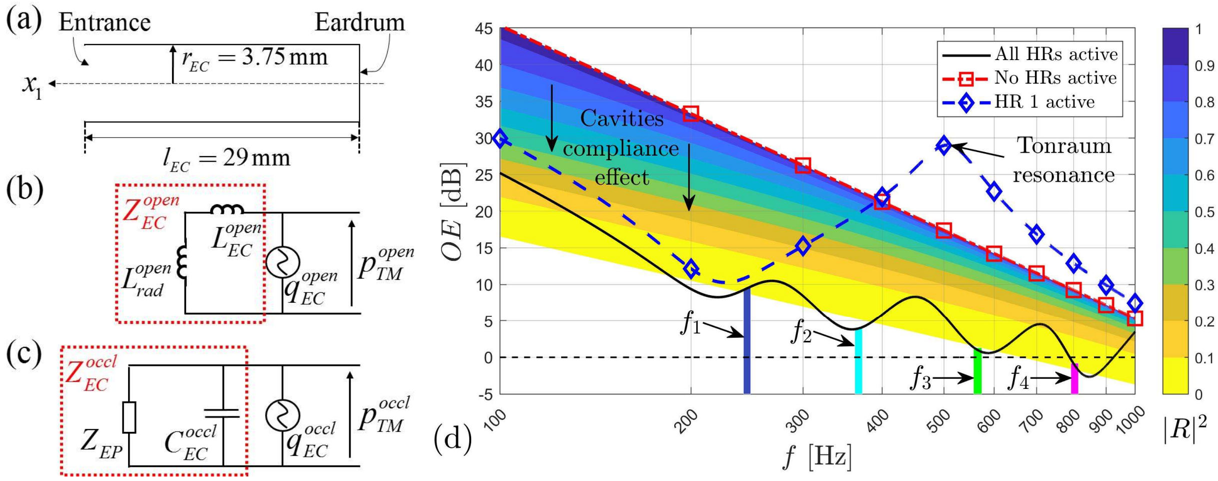

FIG S4: (a) Schematic of the earcanal and associated electro-acoustic model in (b) open and (c) occluded configurations. (d) Occlusion effect (in narrow band) of the meta-earplug with all HRs active, no HRs active and only HR#1 active computed using the electro-acoustic model. Vertical coloured lines indicate frequencies of absorption peaks of the meta-earplug, i.e., $f_1 = 245$ Hz, $f_2 = 367$ Hz, $f_3 = 565$ Hz, and $f_4 = 803$ Hz. Colour-map shows the dependence of the simulated occlusion effect on both the reflection coefficient of the earplug medial surface and the frequency. Note that the cavities compliance effect only applies to frequencies below 200 Hz.

REFERENCES

1. Romero-García, V. *et al.* Perfect and broadband acoustic absorption by critically coupled sub-wavelength resonators. *Sci. Rep.* **6**, 19519 (2016).
2. Romero-García, V., Theocharis, G., Richoux, O. & Pagneux, V. Use of complex frequency plane to design broadband and sub-wavelength absorbers. *J. Acoust. Soc. Am.* **139**, 3395–3403 (2016).
3. Carillo, K., Doutres, O. & Sgard, F. Theoretical investigation of the low frequency fundamental mechanism of the objective occlusion effect induced by bone-conducted stimulation. *J. Acoust. Soc. Am.* **147**, 3476–3489 (2020).
4. Carillo, K., Doutres, O. & Sgard, F. Numerical investigation of the earplug contribution to the low-frequency objective occlusion effect induced by bone-conducted stimulation. *J. Acoust. Soc. Am.* **150**, 2006–2023 (2021).
5. Ang, L. Y. L., Koh, Y. & Lee, H. Plate-type acoustic metamaterials with tonraum resonator for improved sound transmission loss. in *25th International Congress on Sound and Vibration (ICSV25)* (2018).
